# Hemorrhagic bullous eruption with mucosal involvement in a patient with chronic kidney disease

**DOI:** 10.1016/j.jdcr.2026.09.051

**Published:** 2026-10-03

**Authors:** Claudia Spates, John Hanna, Joyce Farah, Daniel Zaccarini, Ramsay Farah

**Affiliations:** a Norton College of Medicine, SUNY Upstate Medical University, Syracuse, New York; b Department of Pathology, SUNY Upstate Medical University, Syracuse, New York; c Department of Dermatology, SUNY Upstate Medical University, Syracuse, New York

**Keywords:** chronic kidney disease, cryptococcus, iododerma, neutrophilic dermatosis, sweet syndrome

## Case presentation

A 76-year-old male with stage IV chronic kidney disease, solitary kidney and coronary artery disease presented to the emergency department for management of symptomatic anemia following a presyncopal episode at home. Home medication regimen included hydralazine 100 mg 3 times per day. Computed tomography angiography of thorax, abdomen, and pelvis was performed for localization of hemorrhage using iohexol 200 mL. Within 48 hours of iodinated contrast administration, patient developed edematous and bullous purpuric plaques with exuberant hemorrhagic crusting. The eruption began on the scalp and neck (Fig 1), and then rapidly progressed to include the trunk, extremities, and face along with periorbital, tongue, epiglottis, and pharyngeal wall edema (Fig 2, Fig 3, Fig 4). Gradual improvement of condition was seen upon administration of hemodialysis (3.5-hour session, 3 times per week over 30 days) and a corticosteroid taper.

**Fig 1:**
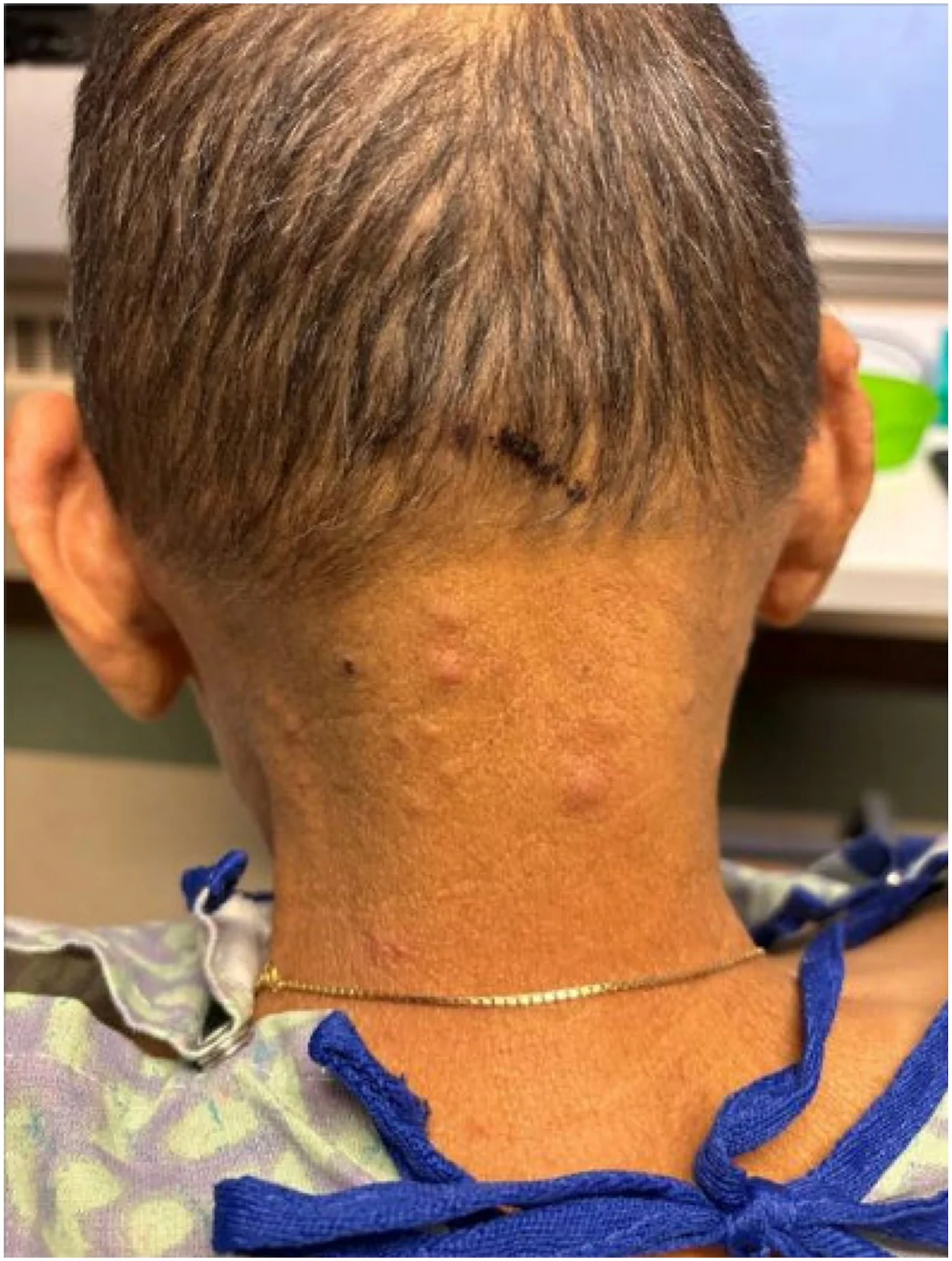
Development of pruritic nodules and hemorrhagic crusting on neck and scalp.

**Fig 2:**
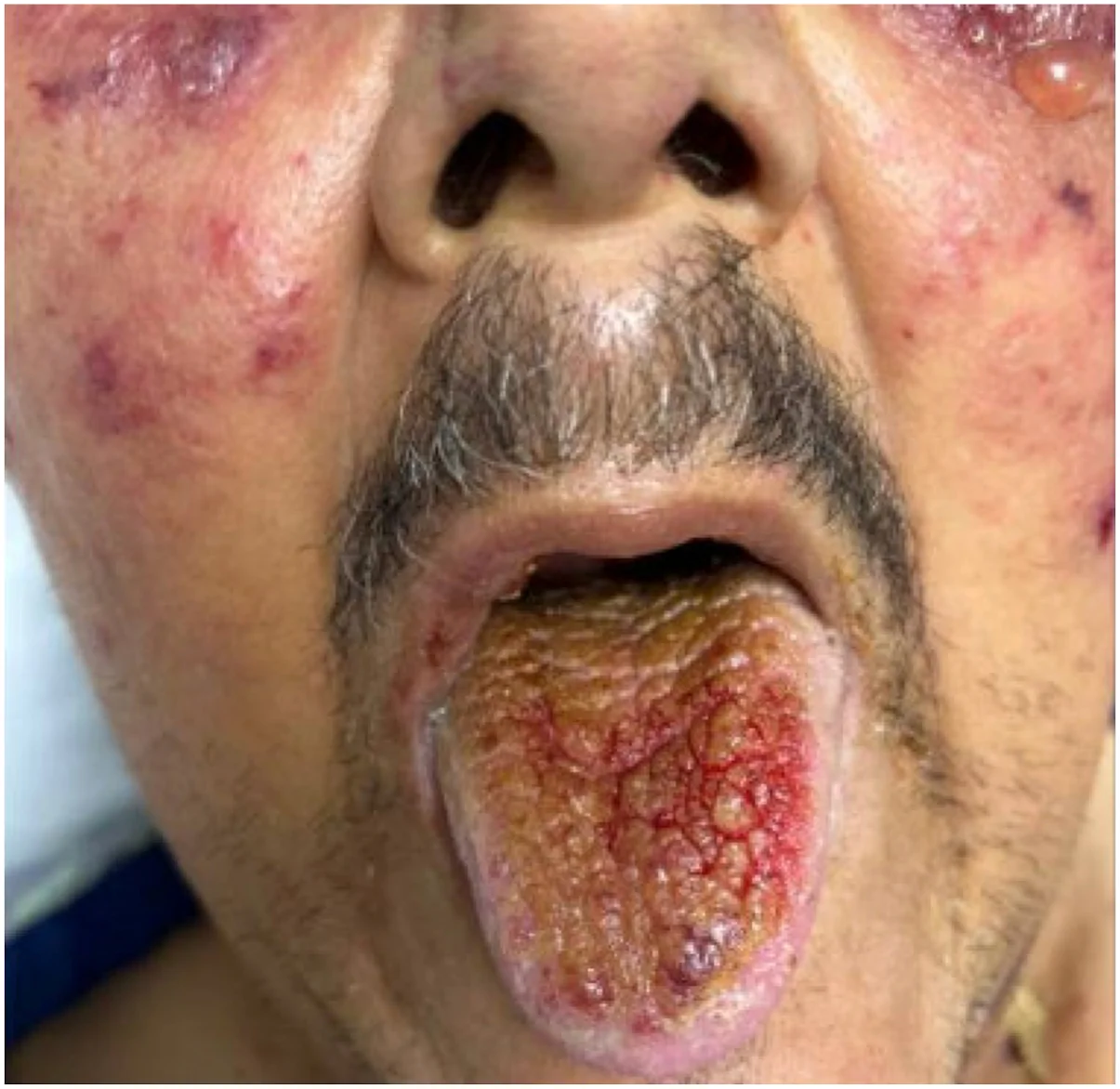
Dorsal tongue surface demonstrated diffuse erythema, erosions, and extensive adherent yellow-brown hemorrhagic crust with prominent fissuring.

**Fig 3:**
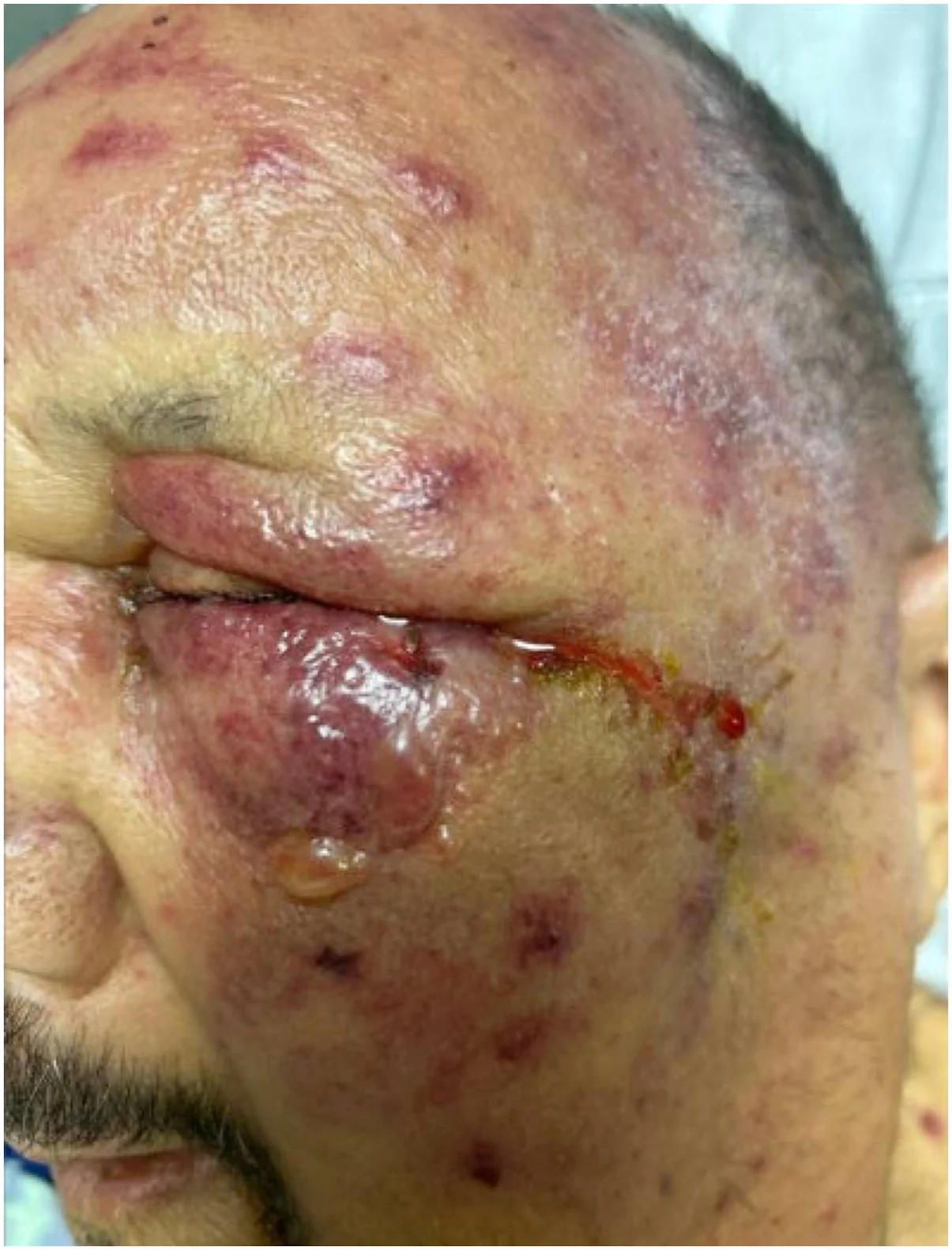
Right periorbital swelling with multiple bullae on lower lids.

**Fig 4:**
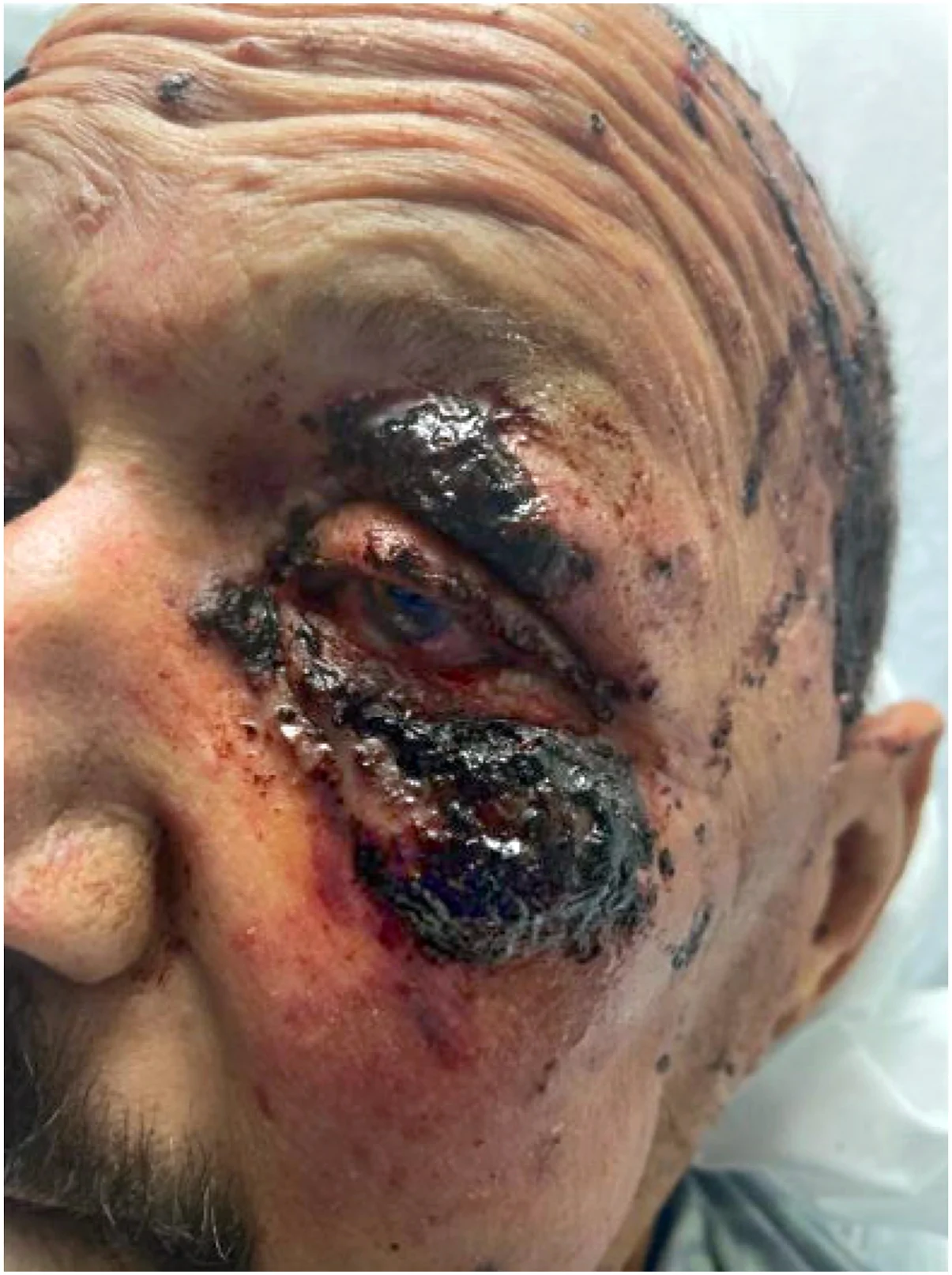
Right periorbital hemorrhagic crusting on both upper and lower lids.

**Question: Which of the following is most helpful in confirming a diagnosis of contrast induced iododerma?**

**A.** Elevated serum or urine iodine.
**B.** Positive Gomori methenamine silver (GMS) stain demonstrating cryptococcal yeast forms.
**C.** Skin biopsy showing granulomatous inflammation.
**D.** Positive antineutrophil cytoplasmic antibodies (ANCA) serology by indirect immunofluorescence.
**E.** Positive Alcian blue stain demonstrating cryptococcal halos.

**Correct Answer: A.** Elevated serum or urine iodine levels are the most specific test for confirming iododerma. Histopathology can be variable and may mimic other conditions. ANCA positivity can occur but is not specific. Positive GMS and Alician Blue stains point to *Cryptococcus* infection.^1^

## Discussion

Iododerma typically presents on face, neck, and chest after initial contrast exposure. However, atypical presentations with oral, ocular, and respiratory mucosa involvement can also occur.

Renal impairment is central to pathogenesis, as reduced iodine clearance in chronic kidney disease leads to systemic accumulation and prolonged exposure to iodine.^1^ Use of hydralazine is also a major risk factor for the development of iododerma through neutrophil activation.

ANCA serology may be positive in iododerma and can mimic ANCA-associated vasculitis or hydralazine-induced lupus/vasculitis. However, ANCA positivity is nonspecific and may reflect neutrophil toxicity associated with iododerma.

Timing of biopsy is critical in identifying the histopathologic picture of iododerma. In acute phase, findings demonstrate dense dermal neutrophilic infiltration with prominent papillary dermal edema, fibrinoid necrosis of vessel walls with leukocytoclasia, and vacuolated cells exhibiting abundant empty cytoplasm and well-defined cell membranes, closely mimicking the capsular halo of *Cryptococcus neoformans* on H&E (Fig 5). Subacute or chronic lesions tend to exhibit pseudoepitheliomatous hyperplasia, neutrophilic microabscesses, granulomatous inflammation, and leukocytoclastic vasculitis, a pattern more likely to suggest an infectious or granulomatous process and 1 that imposes a challenge in diagnosing iododerma. This constellation of findings may overlap with Sweet syndrome, including its histiocytoid variant, and necessitates special stains (GMS, periodic acid–Schiff, and mucicarmine) to exclude true fungal infection.^1^

**Fig 5:**
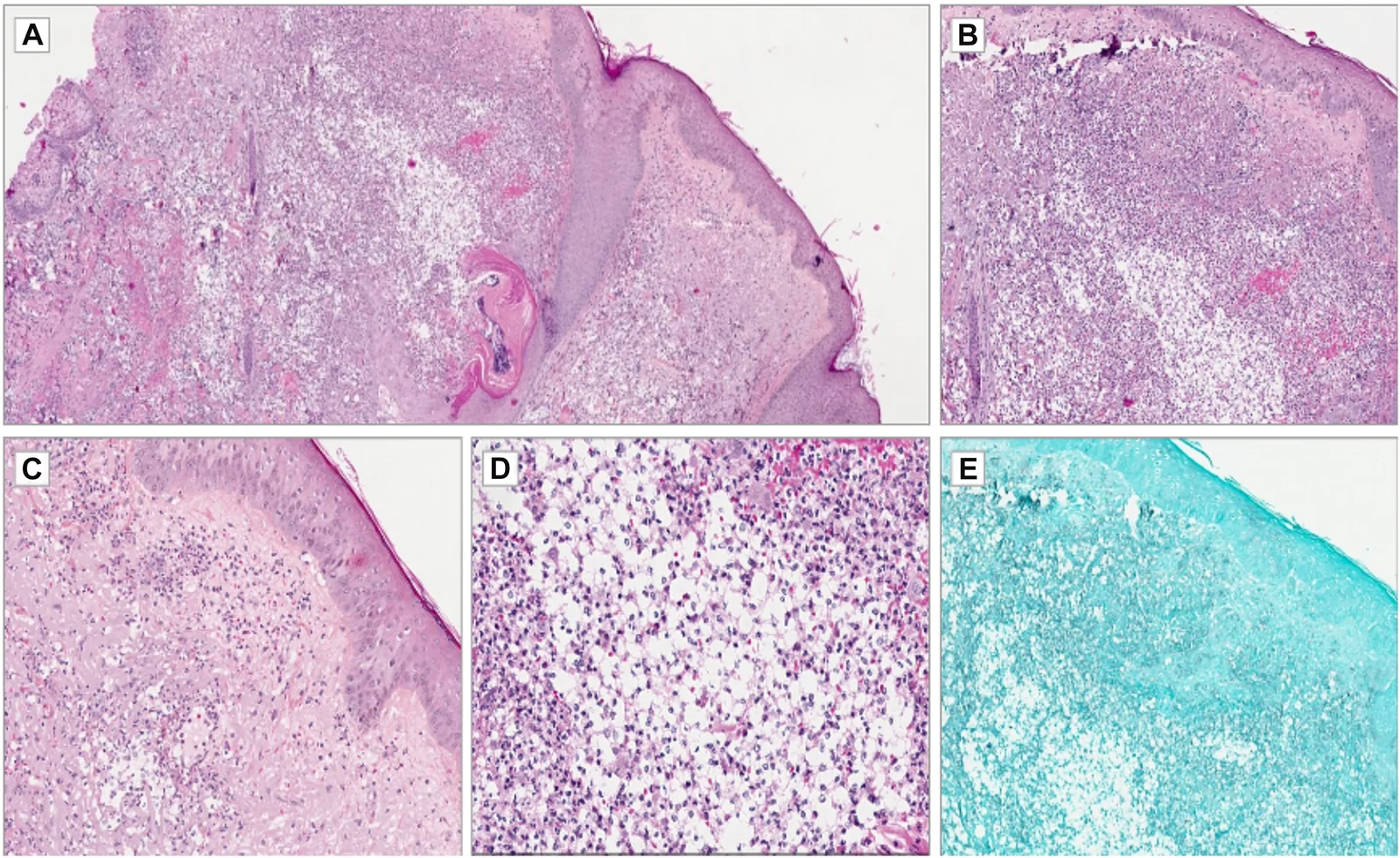
Low-power hematoxylin and eosin (H&E) examination reveals a dense, sheet-like neutrophilic infiltrate diffusely expanding the dermis with papillary dermal edema and involvement of the perifollicular stroma **(A)**. At medium power (H&E), the epidermis is preserved overlying confluent dermal neutrophilia, with pale vacuolated foci displaying cells with cleared-out cytoplasm and a well-defined cell membrane imparting a cryptococcoid halo appearance **(B)**. Foci of fibrinoid necrosis of vessel walls with leukocytoclasia are identified, consistent with a superimposed neutrophilic vasculitis (H&E) **(C)**. At high power (H&E), rounded to ovoid cells with abundant cleared out cytoplasm are seen, closely mimicking the capsular halo of *Cryptococcus neoformans***(D)**. GMS stain is negative for fungal organisms, confirming this as a reactive cryptococcoid change rather than true cryptococcosis **(E)**. *GMS*, Gomori methenamine silver; *H&E*, hematoxylin and eosin.

When presented with cryptococcoid morphology on hematoxylin and eosin and a negative GMS stain, reviewing the patient's clinical history for recent exposure to iodinated contrast media should be a routine consideration. When iododerma is in the differential diagnosis, serum and/or urine iodine levels should be checked early, as elevated levels can provide valuable corroborative evidence and guide management.^1^, ^2^

Hemodialysis holds an important therapeutic role in severe cases of iododerma by enhancing iodine removal and reducing inflammatory burden. Optimal duration and frequency of dialysis depends on the iodine burden, renal function, dialysis efficiency, and whether iodine levels are being monitored.

Prevention relies on strict avoidance of iodinated contrast after diagnosis. Reexposure may result in more severe recurrence. Alternative imaging strategies should be prioritized in patients with chronic kidney disease or prior reactions.

### Declaration of generative AI and AI-assisted technologies in the writing process

During the preparation of this work the author(s) used OpenEvidence AI in order to suggest resources and organize citations. After using this tool/service, the author(s) reviewed and edited the content as needed and take(s) full responsibility for the content of the publication.

## Conflicts of interest

None disclosed.
